# Interplay between the Endocrine System and Immune Cells

**DOI:** 10.1155/2015/986742

**Published:** 2015-08-05

**Authors:** Livia A. Carvalho, Jantje M. Gerdes, Carina Strell, Graham R. Wallace, Joilson O. Martins

**Affiliations:** ^1^UCL Research Department of Epidemiology and Public Health Rm356a, 1-19 Torrington Place, London WC1E 7HB, UK; ^2^Institute for Diabetes and Regeneration Research, Helmholtz Center Munich, Business Campus Garching, Parkring 11, 85748 Garching, Germany; ^3^Department of Oncology-Pathology, Karolinska Institutet, 171 77 Stockholm, Sweden; ^4^Academic Unit of Ophthalmology, School of Immunity and Infection, College of Medical and Dental Sciences, University of Birmingham, Edgbaston, Birmingham B15 2TT, UK; ^5^Department of Clinical and Toxicological Analyses, Faculty of Pharmaceutical Sciences, University of São Paulo, 05508-000 São Paulo, SP, Brazil

Immunoendocrinology is an important field that studies the connection between the immune and endocrine systems. Hormones, including molecules like vitamin D and metabolic components, and neurotransmitters are compounds produced by different cell types that are capable of regulating the cross talk among cells from different tissues. During the last years, it became evident that hormones and neurotransmitters are specific modulators of cells of the immune system by fine-tuning their activation and key functions.

This special issue covers the most recent research elucidating how hormones are able to regulate the recruitment of immune cells to an inflammatory site as well as to explain the hormonal impact on the intracellular signaling cascades that follow after tissue injury. It will cover the mechanisms by which hormones can influence inflammatory process.

Diabetes mellitus is the most common cause of end-stage renal disease and chronic low-grade inflammation is an important factor in the pathogenesis of diabetic complication. Mycophenolate mofetil (MMF) has an anti-inflammatory effect, inhibiting lymphocyte proliferation. J.-W. Seo et al. study the effect of MMF on diabetic nephropathy and investigate its action mechanisms in type 2 diabetic mouse models. They demonstrated that MMF treatment attenuates diabetic nephropathy by decreasing CD4+ T cell infiltration and its related cytokines and chemokines. This anti-inflammatory effect of MMF attenuates podocyte apoptosis independent of glucose control.

MicroRNAs are small noncoding RNA molecules that regulate gene expression in all cell types, which are involved in a wide range of biological processes, exerting functional effects at cell, tissue, and organ levels. G. Ventriglia et al. summarize the most recent data on the potential involvement of microRNAs in autoimmune diabetes and indicate that miRNAs represent major players in the dialogue between beta cells and immune system, regulating several aspects of their function and survival.

Infection with the protozoan parasite* Trypanosoma cruzi* induces a hormonal systemic deregulation. A. F. Nardy et al. illustrated how the sphingosine-1-phosphate (S1P) system regulates both thymic and lymph node T cell egress which is essential for producing and maintaining the recycling T cell repertoire. The presence of this T cell receptor (TCR) bearing cells in the periphery may have potential implications for disease outcome. The authors discuss the possibility that the early egress of undifferentiated CD4−CD8− T cells plays a role in the immunopathologic events in Chagas disease by altering adaptive immune responses.

The current evidence of the role of liver dendritic cells (DC) in the progression from nonalcoholic fatty liver disease (NAFLD) to fibrosis is reviewed by P. Almeda-Valdes et al. Link between lipid metabolism and DC function suggests that immunogenic DC are associated with liver lipid storage, which might represent a possible pathophysiological mechanism in NAFLD.

Interactions between the pineal gland and the immune system have been studied ex vivo. A. P. Herman et al. provide evidence on how interleukin- (IL-) 1*β* suppresses melatonin secretion and its action seems to be targeted on the reduction of pineal gland arylalkylamine-N-acetyltransferase (AA-NAT) protein expression.

Varicocele is the most common cause of infertility in men and the development of varicocele-related testis damage may be caused by disruption of homeostasis between cell proliferation and cell death. In addition, apoptosis is a physiological process by which a sequence of intracellular events results in the programmed elimination of a cell from its environment. Specifically, alterations in the apoptosis of germ cells may be crucial in varicocele-related human infertility and, as a direct consequence, targeting apoptosis may represent an alternative and rational therapeutic strategy in the treatment of varicocele complications. L. Minutoli et al. studied the effect of Polydeoxyribonucleotide (PDRN), an agonist of adenosine A2A receptor, on testicular Neuronal Apoptosis Inhibitory Protein (NAIP) and survivin expression in an experimental model of varicocele. They found that PDRN may represent a rational therapeutic option for accelerating recovery from depressed testicular function through a strategic modulation of apoptosis in experimental varicocele.

